# Compressive Properties of Composite Sandwich Structure with Fractal Tree-Inspired Lattice Core

**DOI:** 10.3390/ma18030606

**Published:** 2025-01-29

**Authors:** Jian Han, Xin Ma, Rui Yang, Shiyong Sun

**Affiliations:** School of Mechanical Engineering, Dalian University of Technology, Dalian 116024, China; jianhan@mail.dlut.edu.cn (J.H.); marsin4517@mail.dlut.edu.cn (X.M.); yangrui@dlut.edu.cn (R.Y.)

**Keywords:** fractal design, lattice structure, mechanical performance, 3D printing, energy absorption

## Abstract

A novel sandwich structure of a fractal tree-like lattice (SSFL) is proposed. The geometry characteristics were constructed based on the fractal tree-like patterns found in many biological structures, such as giant water lilies and dragon blood trees. The compressive performance of the proposed structures with different fractal orders was experimentally and numerically investigated. The experimental samples were made by 3D printing technology. Axial compression tests were conducted to study the compressive performance and failure mode of the SSFLs. The results indicated that the new structure was good at multiple bearing and energy absorption. The finite element method (FEM) was performed to investigate the influence of geometry parameters on the compression behaviors of the SSFLs. The findings of this study provide an effective guide for using the fractal method to design lattice structures with a high bearing capacity.

## 1. Introduction

The composite lattice sandwich structure is an advanced structure known for its high structural efficiency, which integrates material advantages and structural advantages [1]. The lattice sandwich structure is composed of rigid panels and a lightweight lattice core, which are famous for their high specific stiffness, strength, and tensile elasticity [2,3]. Additionally, it offers multifunctional potentials, such as thermal protection and insulation, energy absorption, and noise shields. Incorporating composite material into lattice structures enhances the structure efficiency, as the anisotropic materials can be tailored to maximize properties in each direction [4]. Therefore, composite lattice structures present a synergistic combination of material characteristics and geometry design. 

Composite lattice structures have attracted increasing attention and research in recent years and various lattice structure topologies have been proposed. Common methods for composite lattice manufacturing include woven wire [5], co-cured corrugated lattice cores [6], snap-fitting [7], and 3D printing [8]. Khaledi et al. [9] experimentally determined the mechanical response of the composite sandwich structures with an M-shaped core, which was subjected to three-point bending loads to investigate the flexural strength. Wang et al. [10] designed a double-layer woven lattice structure which was manufactured by a vacuum bag molding process. In-field explosion experiments revealed that the structure had excellent blast resistance and EM wave transmission characteristics. Xu et al. [11] used long-fiber-reinforced thermoplastic composite parts manufactured through injection molding to create an octet lattice structure. Out-of-plane compression tests revealed that the mechanical properties of the structure were sensitive to the presence of the connections. Lee et al. [12] developed a composite lattice structure that they termed “semi-wire-woven bulk Kagome”, combining woven wire with epoxy resin fillings. This structure showed a performance comparable to honeycomb cores and pyramidal CFRP lattices. Wang et al. [13] designed an X-shaped lattice core using the hot-press technique. Dong et al. [14] explored a simple “snap-fitting” assembly method for fabricating octet-truss cellular materials from CFRP laminate materials. Zhang et al. [15] proposed a hybrid concept to design the lattice structure manufactured by snap-fitting the CFRP out-of-plane trusses and Ti-6Al-4V in-plane trusses. Furthermore, the application of 3D printing has expanded the geometric design possibilities of lattice sandwich structures. Chen et al. [16,17] utilized the snap-fitting method to fabricate fused-deposition-modeled body-centered cubic (BCC), face-centered cubic (FCC), and octet lattices, with filaments deposited along the struts. This approach significantly enhanced both the surface quality and the printing efficiency of 3D printing technology. Ye et al. [18] determined the failure mode of a lattice sandwich structure with the column cores integrally manufactured by 3D printing, observing no damage to the joint between the core and face sheet. Lei et al. [19] proposed a conformal sandwich lattice design strategy. The elastic properties of the conformal sandwich lattice can be widely user-specified from 109.0% to 226.1%. Fan et al. [20] developed a graded porous lattice core sandwich structure utilizing 3D printing techniques to implement precise porosity control and achieve a balance between lightweight and high-mechanical-performance materials.

The sandwich structure of the pyramid lattice (SSPL) is a classical composite lattice configuration known for its excellent balance of compressive and shear mechanical properties [21,22,23]. The failure of pyramid lattice structures is often brittle, typically resulting from the buckling of struts or fractures at the core–panel joints. The uniaxial compressive stress–strain curve of an SSPL usually drops abruptly, implying a limited capacity for energy absorption. The energy absorption capacity of composite lattice structures is just as crucial as their compressive strength. Researchers have done a lot of work to strengthen the mechanical capacity of SSPLs [24]. Fan et al. [25] put forward the adoption of a new hierarchical material reinforced by a woven textile sandwich composite to optimize the mass efficiency and specific energy absorption of SSPLs. However, the performance of the woven textile sandwich is not uniform, and the performance stability is not guaranteed. Zhao et al. [26] proposed a new preparation process that combined a carbon fiber strut and the low-modulus foam sandwich structure to improve the mechanical properties of SSPLs; however, the holes in the panel needed to be prefabricated to stitch the strut, which weakened the strength of the panel. Li et al. [27] adopted an aluminum-alloy-reinforced frame to enhance the panel–core strength, which invisibly increased the quality of the structure as well as the density of the SSPL. Hu et al. [28] proposed a novel panel–core connection technology based on the hot-melting characteristic of CF/PEEK composites. The results of the low-velocity impact test showed that panel–core strength was indeed enhanced, and the failure mode was brittle fractures that occurred in the struts.

In this paper, drawing inspiration from the fractal tree, a lattice core sandwich structure with secondary bearing capacity and energy absorption capacity is designed. Fractals are a mathematical concept and fractal theory has been widely studied in biology, chemistry, materials science, and other fields [29,30,31,32]. Fractals can also be used to design structures with mechanical properties desirable for engineering projects, such as foam structures with tailored density distributions [33], crash-resistant structures [34], energy-absorbing structures [35], and mechanical metamaterials with a Poisson’s ratio of zero [36]. The fractal tree is a typical fractal geometry and fractal tree-like branching structures can be found in nature, such as in dragon blood trees or giant water lilies [37]. This structural feature helps the plants to withstand both bending and axial loads due to strong wind force or their weight. The tree-like branching structures can be efficiently used in civil engineering and architecture to withstand load and absorb energy, such as the supporting struts used for shed roofs in some large buildings [38]. Coincidentally, the sandwich structure of a lattice is similar to these structures at different scales, and both bending and axial load-bearing capacities are concerned in the design and application of lattice structures. Accordingly, a sandwich structure with a fractal lattice core (SSFL) mimicking the natural tree-like structures was proposed and investigated in this study. The lattice structure was designed according to the characteristics of fractal tree geometry and manufactured by 3D printing technology. The finite element (FE) models were constructed to investigate the load-bearing capacity of the proposed structure with different geometric parameters.

## 2. Design and Manufacturing 

### 2.1. Geometric Models

**Figure 1:**
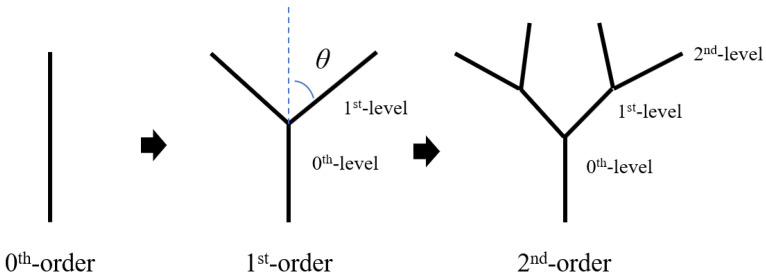
Evolution of the binary tree. Reprinted with permission from Ref. [39]. 2021, Elsevier Ltd.

From a geometric point of view, an arbitrary, complex, tree-like fractal structure is generated by iterative function systems. One of the most common fractal tree models is the symmetric binary tree [39]. As shown in Figure 1, the vertical line segment of length 1 is used as the trunk, and the top part is bifurcated into two branches. The angle between each branch and the extension line of the trunk is *θ* (0° < *θ* < 180°), and the length of each branch is *r* (0 < *r* < 1). These two branches, in turn, form the backbone of the subtree, which in turn divides into two smaller branches according to the same rules. The angle is still *θ* and the length of the four new branches is *r*_2_. Adding smaller branches according to this rule results in a symmetric binary tree.

**Figure 2:**
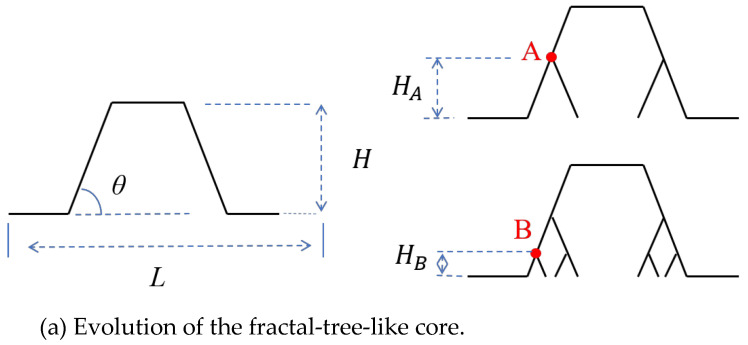

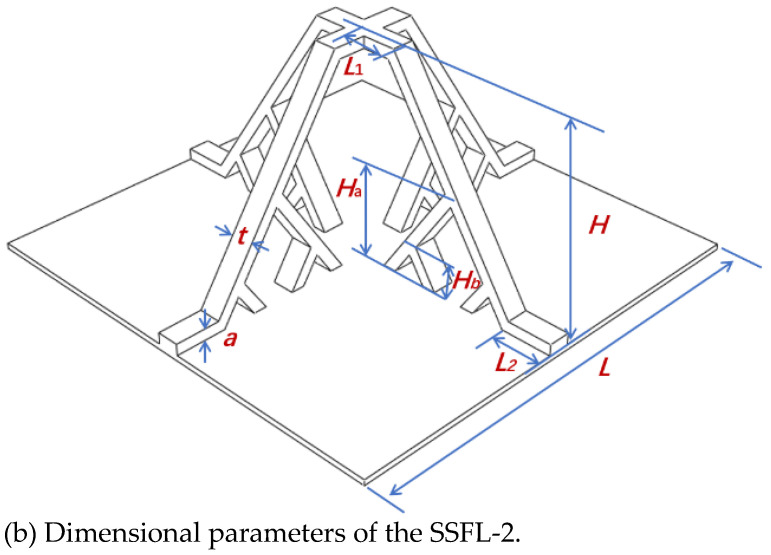
Geometric design of the fractal lattice core.

In this paper, the SSFL is designed in a fractal way based on a traditional pyramid lattice core. The SSFL model construction process is as follows:

The pyramid lattice core consists of four oblique struts, which can be seen as the first four trunks. The key parameters for the fractal tree are *θ* and *r*. In the design of the SSFL, to maintain the symmetry of the structure, *θ* is consistent with the inclination angle of a pyramidal strut, and r is a subordinate parameter determined by the bifurcation positions A and B, as shown in Figure 2a. The distance between the upper and lower panels is denoted as *H*. The 1-level bifurcation is introduced from point A on the strut, *H_A_*/*H = r*_1_; and the 2-level bifurcation is introduced at point B, *H_A_*/*H_B_* = *r*_2_. Thus, first-order lattice (SSFL-1) and second-order lattice (SSFL-2) structures are formed due to the respective bifurcation level. The geometric characteristics of the SSFL-2 are illustrated in Figure 2b. The single length of a cell is *L*. The thickness and height of the strut cross-section are denoted as *a* and *t,* respectively. 

The relative density formula of the fractal lattice structure is denoted as follows:

(1) $${\bar{\rho }}_{0}=\frac{4\cdot a\cdot t\cdot [L_{1}+L_{2}+\sqrt{(\frac{L}{2}-L_{1}+L_{2})+H^{2}}]}{H\cdot L^{2}}$$

(2) $${\bar{\rho }}_{1}=\frac{4\cdot a\cdot t\cdot [L_{1}+L_{2}+(\frac{H_{A}}{H}+1)\sqrt{\left(\frac{L}{2}-L_{1}+L_{2})+H^{2}\right)}]}{H\cdot L^{2}}$$

(3) $${\bar{\rho }}_{2}=\frac{4\cdot a\cdot t\cdot [L_{1}+L_{2}+(\frac{H_{A}}{H}+\frac{H_{B}}{H}+1)\sqrt{\left(\frac{L}{2}-L_{1}+L_{2})+H^{2}\right)}]}{H\cdot L^{2}}$$

It is worth noting that the structure proposed in this paper can be manufactured by 3D printing, as well as other traditional manufacturing methods due to its two-dimensional design features, such as by reasonable process design.

### 2.2. Lattice Fabrication and Compression Test

**Figure 3:**
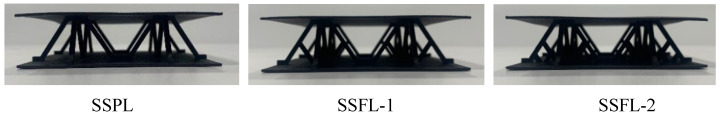
Test specimens.

In this study, structure samples and material test samples were drawn using 3D SolidWorks Premium 2019 SP0.0 and printed through selective laser sintering (SLS) using an HP Jet Fusion 4200 3D printer. The raw material for 3D printing was Polyamide(PA12) with a density of *ρ* = 1.15 g/cm^3^. Both the material and the printer were supplied by Hewlett-Packard Development Company, L.P., located in Palo Alto, CA, USA. The critical dimensions of the parameters and test specimens are shown in Table 1 and Figure 3, respectively. The uniaxial compressions were carried out according to the ASTM C365M-05 Standard [40] by the universal testing machine WDW-20E, Shidaishijin Instrument Co., Jinan, China; the loading speed of the test was 1 mm/min. 

### 2.3. Finite Element Method Simulation

**Figure 4:**
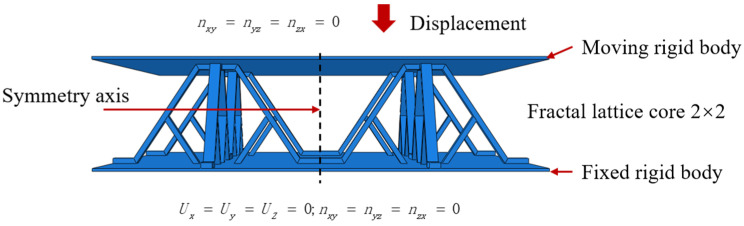
Quasi-static compression finite element model of SSFL (SSFL-1 as an example).

The finite element method (FEM) is conducted based on the commercial finite element software ABAQUS/Standard 2017 to predict the deformation process of the SSPL and SSFLs under quasi-static compression. Solid Element C3D8R was selected to simulate the structure. The panels were set to the rigid body. The lower panel was set to be completely fixed, where U_x_, U_y,_ and U_z_ are the displacements of the fixed rigid plate in the three directions of x, y, and z, and n_xy_, n_yz_, and n_zx_ are the three rotational degrees of freedom, as shown in Figure 4. The upper panel was set to be fixed in n_xy_, n_yz_, and n_zx_. Displacement loads and lateral disturbances were applied on the upper panel. The rigid plate retained its shape and size during movement and load. Considering the symmetry of the lattice structure, 1/4 of the structure was taken to be simulated. The compression simulation of each of the three structures under the same conditions was carried out, respectively.

**Figure 5:**
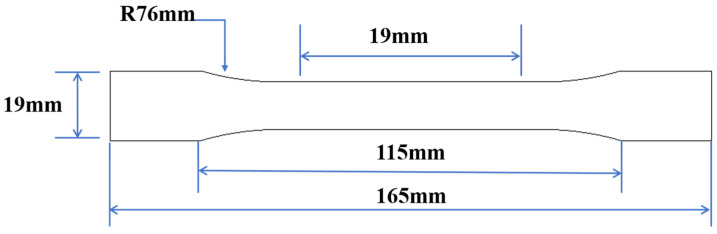
The critical dimensions of nylon dog-bone.

The nylon material can be considered a homogeneous isotropic material, and the Poisson ratio is 0.3. To obtain precise finite simulation results, five dog-bone tensile specimens with the same fabrication process as the lattice samples were prepared to obtain the mechanical parameters of the matrix material. The tensile behavior of the matrix materials was tested on WDW-20E at 1 mm/min by a standard tensile test according to the ASTM D638-14 Standard [41] using the Type 1 geometry, in which the critical dimensions are shown in Figure 5. Considering the post-yield effect of dog-bone specimens, the nominal stress–strain data were converted into true stress–strain data with Equations (4) and (5) to characterize the plastic behavior of nylon material. The average Young modulus of three specimens was 1710 MPa and the true plastic stress–strain data are shown in Table 2.

(4) $$\sigma_{true}=\sigma_{norm}(1+\varepsilon_{norm})$$

(5) $$\varepsilon_{true}=\ln(1+\varepsilon_{norm})$$

General contact was selected between the panels and the core. The contact surface between the rigid body and the lattice structure was set to the general contact mode. The large deformation switch was turned on because the compression process of the structure was nonlinear.

## 3. Results and Discussion

### 3.1. Compressive Performance of Structures 

**Figure 6:**
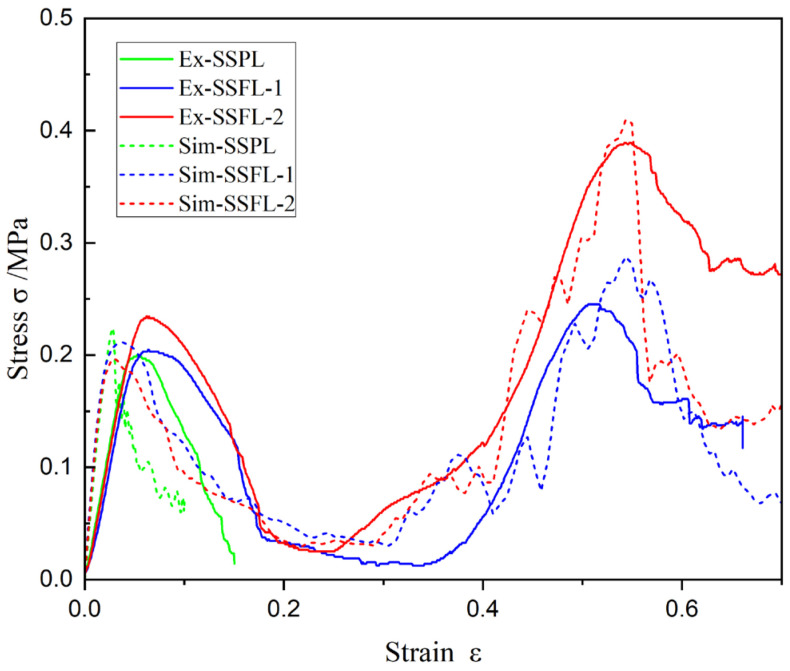
Stress–strain response of SSFLs as measured experimentally and predicted by finite element model.

**Figure 7:**
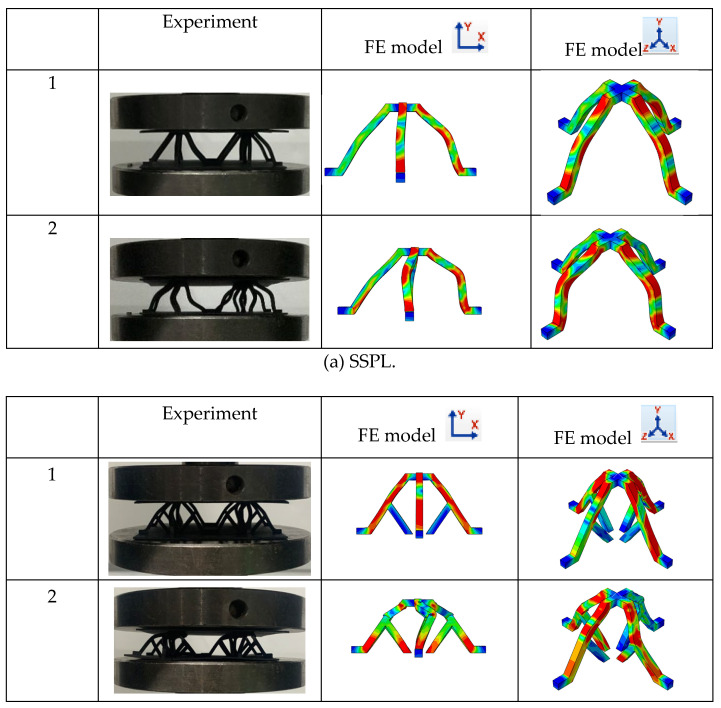

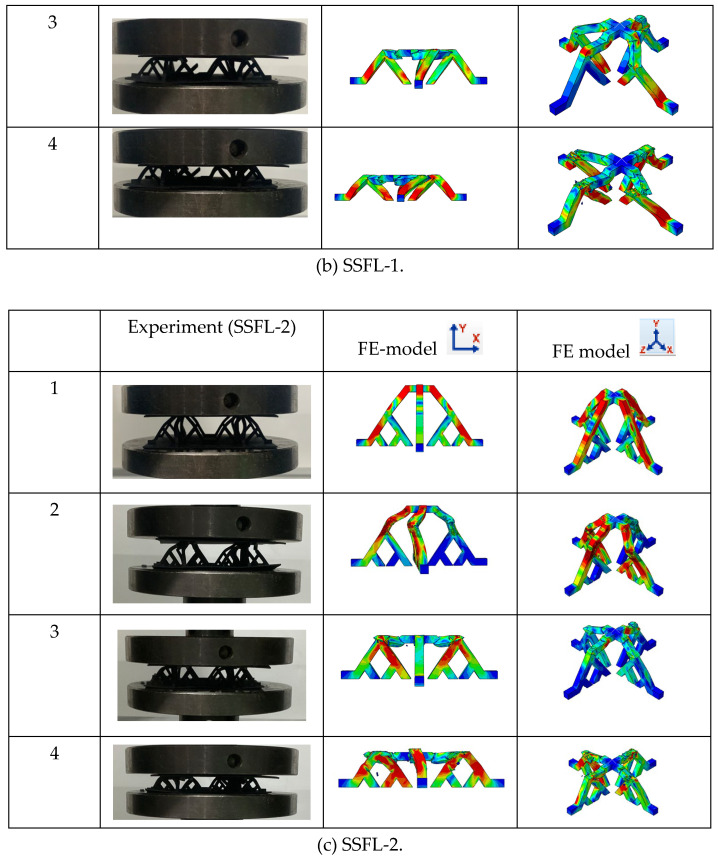
Deformation mechanism of SSFLs at various strain levels as measured experimentally and predicted by finite element model: (**a**) SSPL, (**b**) SSFL-1, and (**c**) SSFL-2. The Abaqus contour plot is used to visualize the simulation results of the structures. It uses different colors to show the distribution of specific stresses within the model. In this illustration, red indicates that the stresses in the structure have reached the limits of the material, blue indicates zero stress, and green indicates an intermediate stress state.

Figure 6 and Figure 7 show the stress–strain curves and the deformation modes of the SSPL and SSFLs under the uniaxial compression load, respectively. The deformation mode of the SSPL is simple: when the stress in the strut reaches the yield strength of the PA material, the four struts begin to bend at their midpoint, as shown in Figure 7a. Once the strain exceeds the limit of the material, fractures occur, resulting in the release of energy. This phenomenon is reflected in the stress–strain curve as a rapid increase followed by a sharp decrease. The deformation modes of the SSFL-1 and SSFL-2 are similar. As the displacement of the indenter increases, the trunks of the SSFLs begin to deform once the load exceeds the bearing capacity. Due to variations in printing accuracy and structural defects, the stress distribution among the four struts is not uniform, leading to asynchronous deformation. From Figure 7b,c, it can be observed that the deformation of the struts causes the slippage of the upper panel, resulting in different failure modes for the four struts. One strut fractures in the middle of the tree trunk, while the opposite strut bends towards the direction of the upper panel’s slip. The other two opposite struts bend laterally, and cracks appear at the junction between the trunks and the branches. As strain increases, stress declines and remains low for a period. The upper panel continues to descend until it contacts the first-order struts, while both the first-order and second-order tree-branch-like struts take on a bearing role in the secondary stage. At this point, the initial bearing strength is exceeded. Notably, the secondary strength of the SSFL-2 is significantly higher than that of the SSFL-1. This difference arises because the slippage of the upper panel and the deformation of the branch struts during the initial bearing stage make it easier for the branch parts of the SSFL-1 to bend laterally. In contrast, the SSFL-2 has a more structurally complete secondary branch with a high elastic modulus, which provides greater resistance to deformation, allowing it to maintain a relatively vertical position for subsequent bearing.

The failure modes of all structures in this study do not exhibit buckling failures. Compliance is a criterion for determining whether buckling has occurred in a strut. The compliance can be calculated through Equation (6). If a strut of the SSPL happens to buckle, the buckling strength is represented as in Equation (8) [15].

(6) $$\lambda =\frac{\mu \cdot L_{0}}{i}=\frac{\mu \cdot \sqrt{H^{2}+(\frac{L}{2}-L_{1}-L_{2})}}{i}$$

(7) $$i=\sqrt{\frac{I_{0}}{A_{0}}}$$

(8) $$\sigma =4\cdot \frac{\pi^{2}\cdot E}{\lambda^{2}}$$

where *μ* is the length factor, *L*_0_ is the length of a strut, and *i* is the inertial radius. Additionally, *I*_0_ and *A*_0_ are the torque and cross-sectional area of the strut, respectively, while *E* is the elastic modulus of the PA material. The cross-section of the strut is rectangular with 2 mm × 2 mm and is fixed at both ends. The compliance value of the SSPL strut is 18.74. The critical compliance values for buckling failure are determined as when the critical buckling stress of the strut equals the yield strength of the material. The critical compliance value *λ_p_* is shown in Equation (9).

(9) $$\lambda_{P}=\sqrt{\frac{\pi^{2}E}{\sigma_{p}}}$$

where *σ_p_* is the yield strength of the PA material. The critical compliance value of the structure is 23.55, which is greater than the actual compliance value of 18.74. Consequently, the failure mode of the SSPL is identified as bending failure. For the SSFL-1 and SSFL-2, the presence of branches further shortens *L*_0_, which decreases compliance, and thus, buckling failure does not occur. Therefore, the fracture strength of the SSPL can be calculated from the cross-sectional area of the bar, as shown in Equations (10) and (11).

(10) $$\sigma =\frac{4\cdot A_{0}\cdot \sin\theta }{L^{2}}\cdot \sigma_{p}$$

(11) $$\sin\theta =\frac{H}{\sqrt{{\left(H\right)}^{2}+{\left(\frac{L}{2}-L_{1}-L_{2}\right)}^{2}}}$$

where *θ* is the inclination angle of the member strut and *L* is the length of the square panels. For the SSFLs, the fracture strength can be calculated using the same equation. It is important to highlight that the load-bearing capacity of the branch section should be twice that of the trunk section, as the number of struts supporting it is theoretically doubled. This implies that the secondary strength would be twice the initial strength. When comparing experimental results to theoretical compression strength, the results for the SSFL-2 align more closely with the strength equation mentioned earlier, as shown in Table 3. The differences between the individual experimental results and the theoretical predictions are minimal and can be utilized for forecasting purposes. However, the secondary bearing strength for the SSFL-1 shows a significant deviation from the test results due to the manufacturing error.

### 3.2. Energy Absorption Performance Analysis

Total energy absorption is the amount of energy absorbed by the structure during deformation, and it is calculated as follows [42]:

(12) $$E_{A}=\int_{0}^{\delta }F(x)dx$$

where *δ* is the deformation displacement of the structure and *F(x)* is the instantaneous load during compression. For the SSPL, *δ* is 2; for the SSFLs, *δ* is 12. Specific energy absorption (*SE*) is defined as the energy absorption per unit mass of a structure, as shown in Equation (13). Unit volume energy absorption (*UVE*) is defined as the energy absorption per unit volume of a structure, as shown in Equation (14).

(13) $$SE=\frac{E_{A}}{\rho }$$

(14) $$UVE=\frac{E_{A}}{U}$$

where *ρ* is the density of the structure and *U* is the volume of the structure. The greater the specific energy absorption and unit volume energy absorption, the better the energy absorption performance of the structure. Table 4 shows that the energy absorption and specific energy absorption performance of the structure are enhanced with increasing fractal order. The SSFL-2 has the strongest ability to absorb energy, and its energy absorption is 10.6 times that of the SSPL and 1.6 times that of the SSFL-1. In terms of specific energy absorption, the SSFL-1 is three times that of the SSPL, and the SSFL-2 is four times that of the SSPL. The energy absorption capacity of this structure is fully proven. 

### 3.3. Comparison Between the Experiment and Simulated Results

Figure 7 shows the comparison of the test and simulated compression performances of the SSPL and SSFLs. During the compression of the strut, fractures and lateral instability occur, and their positions align with what was observed experimentally. The deformation contour diagram reveals that the sliding of the upper panel causes the secondary structure to bear loads asymmetrically; some secondary struts bend inward while others bend laterally. This lateral bending diminishes the load-bearing capacity of the struts, preventing the secondary structure from achieving its full potential. For the SSFL-2, a second-order fractal significantly enhances the stiffness of the branch section, resulting in minimal lateral bending during the initial bearing stage. Moreover, after the trunk section fails, the branch section can return to its original position and maintain its load-bearing capacity.

Additionally, the numerical results and discrepancies between the simulations and experiments are detailed in Figure 7 and Table 3. The simulated strain–stress curves align well with the experimental results, including their trends and peaks. The maximum simulation error is the initial bearing strength of the SSFL-2, namely 1.7%. Therefore, the finite element method is effective for simulating the compression performances and failure modes of lattice structures.

## 4. Parameter Analysis

As previously discussed, incorporating fractal design can improve the mechanical properties of a pyramidal structure. Therefore, it is crucial to examine how geometric parameters affect this structure. This section will focus on the impact of the strut cross-section and the position of the first-order bifurcation on the overall structure using the finite element method, assuming that all structures are ideal and do not contain any additional perturbations.

### 4.1. Effect of Cross-Section

Four groups of SSPLs and SSFLs are established in different cross-sections by changing the dimensions a and t: Group A (a = 1, t = 1); GB (a = 1, t = 2); GC (a = 2, t = 1); and GD (a = 2, t = 2). The designators are shown in Table 5.

**Figure 8:**
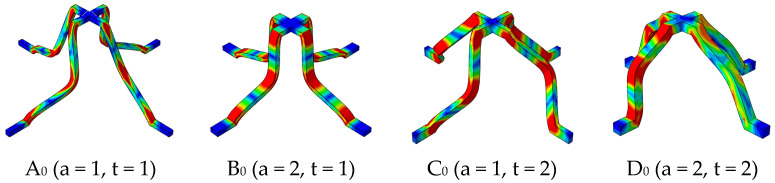
The deformation modes of SSPLs in different cross-sections.

Figure 8 shows the deformation modes of the SSPLs. The failure modes of A_0_, B_0_, and C_0_ are buckling and D_0_ is bending of the struts, consistent with the analysis in Section 3.1. The strut will be unstable in the direction where the compliance value is smaller, so B_0_ buckles inwards to the panel and C_0_ buckles laterally.

**Figure 9:**
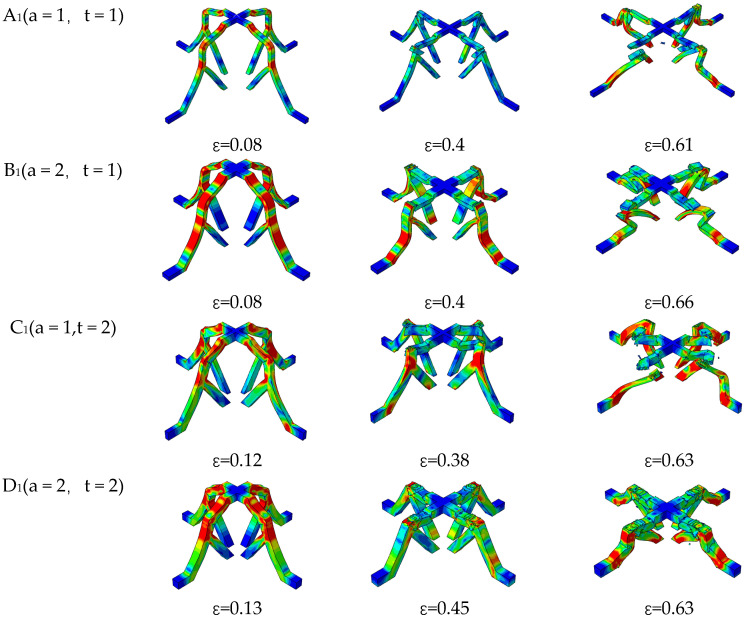
The deformation modes of the SSFL-1s in different cross-sections.

The initial deformation and failure modes of the SSFL-1 structures are quite similar, primarily involving the bending and fracturing of the trunk sections, as shown in Figure 9. Notable differences occur with the increase in uniaxial compression. The trunk section of A_1_ is broken twice due to its large proportion. One break occurs in the middle of the trunk and another at the bifurcation point. However, the integrity of the branch is not affected, as in the case of D_1_. In the case of the other two structures, B1 and C1, the entire structures are subjected to the initial compressive force. The trunk breaks and the branches deform simultaneously. The deformation characteristic of B_1_ is the inward bending of the outer branches, and C_1_ exhibits the lateral bending of all branches, consistent with B_0_ and C_0_, respectively. These deformations undermine the integrity of the branch parts and diminish their bearing capacity. As the displacement increases, only A_1_ and D_1_ are complete structures able to play the bearing role, while the deformation of B_1_ and C_1_ becomes more pronounced.

**Figure 10:**
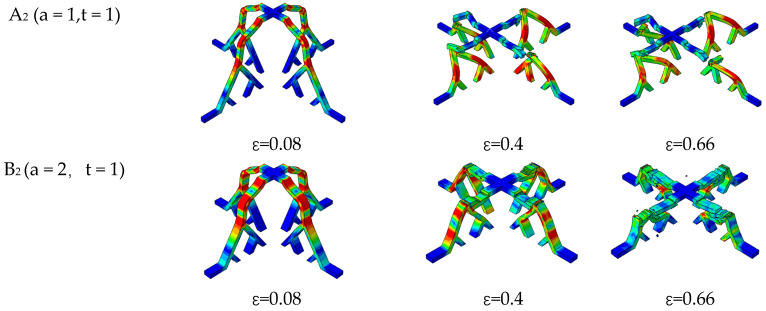

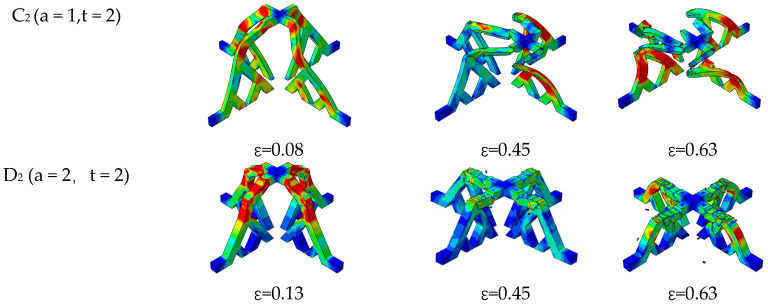
The deformation modes of the SSFL-2s in different cross-sections.

The initial deformation and failure modes of the SSFL-2s are similar to those of the SSFL-1s, as shown in Figure 10. In the following deformation process, A_2_ and B_2_ exhibit different responses compared to A_1_ and B_1_. As the branch section begins to bear the load, A_2_ also experiences lateral bending due to the strengthening effect of the secondary fractal on the in-plane stiffness of the branch part being bigger than that on the out-plane stiffness. Meanwhile, the inward bending of B_2_ is reduced compared to B_1_, which means the second-order fractal design is more effective for Group B. As displacement increases, the deformation of all structures also increases, while the secondary branch section remains stable. The deformation of C_2_ is greater than that of C_1,_ while the difference between D_1_ and D_2_ is quite small.

**Figure 11:**
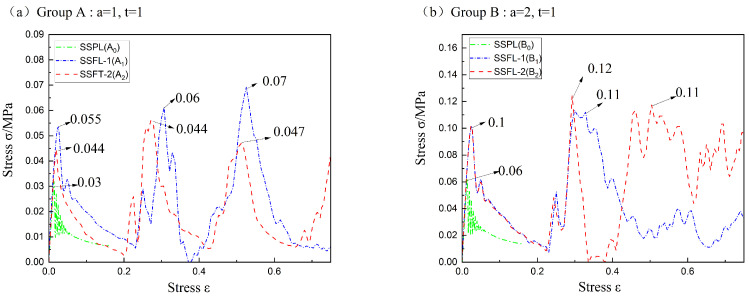

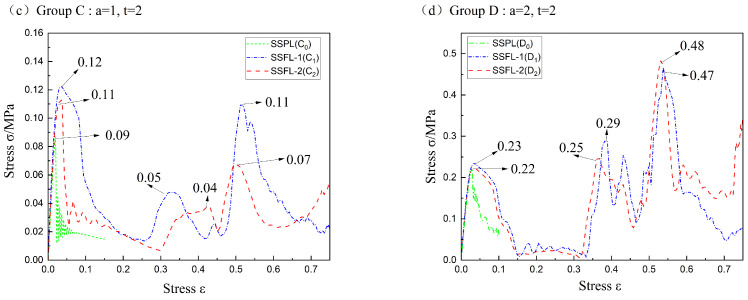
The stress–strain curves of the SSPLs and SSFLs in different cross-sections: (**a**) comparison for group A (a = 1, t = 1); (**b**) comparison for group B (a = 2, t = 1); (**c**) comparison for group C (a = 1, t = 2); (**d**) comparison for group D (a = 2, t = 2).

From the comparison of the strain–stress curves (Figure 11), it can be found that the overall mechanical properties of the SSPL are improved by fractal design in all groups. The SSFLs have multiple bearing capacities and even higher bearing strength in some conditions. In Groups A, B, and C, the initial bearing strengths of the SSFL-1s are 2, 1.7, and 1.4 times higher than the SSPLs. In Group D, the secondary bearing strengths of the SSFL-1 and SSFL-2 are over two times higher than that of the SSPL. 

By a comparison of strength, A_1_ has a better bearing capacity than A_2_. B_2_ overcomes the premature deformation of the branch section before bearing and thus obtains a better bearing capacity than B_1_. The lateral bending of C_1_ and C_2_ causes the secondary strength to be lower than the initial strength. The strength of C_1_ is significantly larger than C_2_ due to a lesser degree of lateral bending.

**Figure 12:**
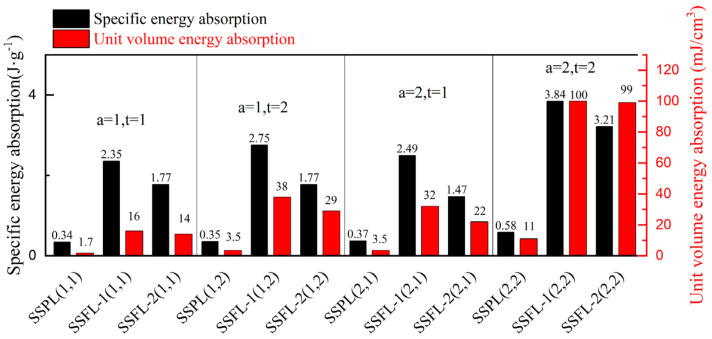
Comparison of specific energy absorption and unit volume energy absorption of SSPLs and SSFLs in different cross-sections.

The energy absorption of the SSFL-1 consistently ranks highest in the four groups, as shown in Figure 12. In addition, the calculation range of the strain is 0–0.7. Once the strain exceeds 0.7, the SSFL-2 enters the densification stage earlier than the SSFL-1 and its energy absorption capacity increases significantly.

As a result, SSPLs with slender struts can achieve relatively large enhancements through fractal design, while the effect of the cross-section shape cannot be neglected. For the same cross-sectional area, the structure obtained by choosing a cross-section of a > t for the fractal is better in terms of strength and energy absorption. The secondary strength of the SSFLs with thick struts is greatly enhanced by fractal design. Additionally, a first-order design is sufficient to optimize the SSPL without considering manufacturing defects in Groups A, C, and D.

### 4.2. Effect of H_A_/H 

In this section, the SSFLs of two cross-section sizes with different ratios of H_A_/H are discussed. One is (a = 2, t = 1), which is sensitive to the fractal design both in strength and energy absorption, and another is (a = 2, t = 2), which is insensitive to fractal design in strength. During the analysis, different ratios of H_A_/H are obtained by changing the value of H, while H_A_ remains constant.

**Figure 13:**
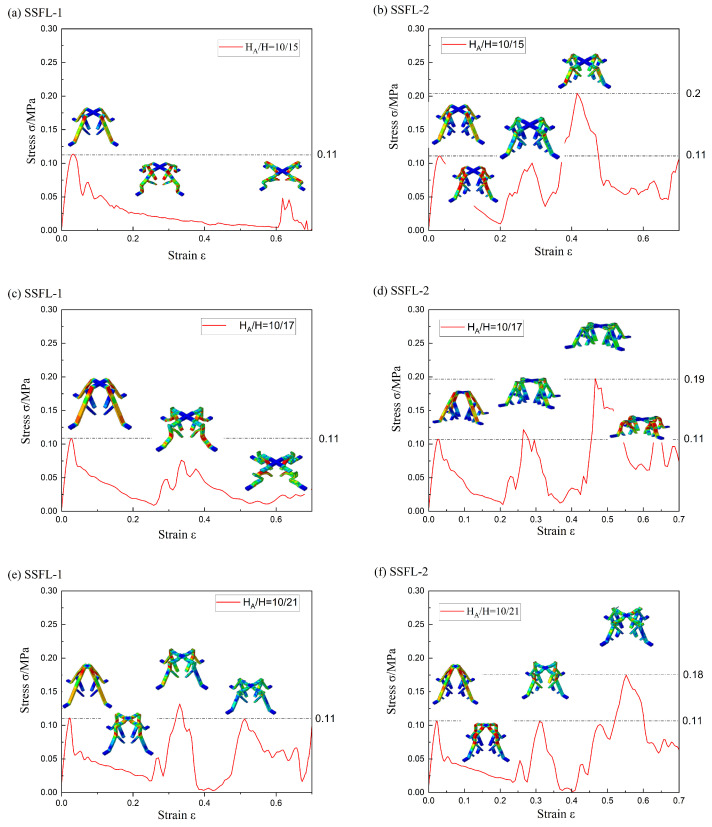
The deformation and stress–strain curves of the SSFLs: (a = 2, t = 1) in different H_A_/H. (**a**) SSFL-1 in H_A_/H = 10/15; (**b**) SSFL-2 in H_A_/H = 10/15; (**c**) SSFL-1 in H_A_/H = 10/17; (**d**) SSFL-2 in H_A_/H = 10/17; (**e**) SSFL-1 in H_A_/H = 10/21; (**f**) SSFL-2 in H_A_/H = 10/21.

The SSFL-1 with the cross-section size of (a = 2, t = 1) is significantly influenced by the bifurcation position, as shown in Figure 13. When H_A_/H = 10/15, the bifurcation position is in the upper part of the trunk. The bending occurs firstly in the middle of the outer branches, instead of in the trunk part like the other SSFLs mentioned above, leading to severe deformation and an almost complete loss of secondary bearing capacity. With the increase in H, the deformation modes change due to the extension of the trunk length, and the secondary strength of the SSFL-1 is improved.

All the SSFL-2s have a tertiary bearing capacity due to the secondary fractal design and the third strength is the highest. This is because the secondary bifurcation adds a node to the branch part and improves the stiffness of the branch portion of the tree. Thus, the fracture occurs in the relatively softer trunk part, while the branch portion remains largely intact after the trunk fractures, allowing it to carry the load again.

**Figure 14:**
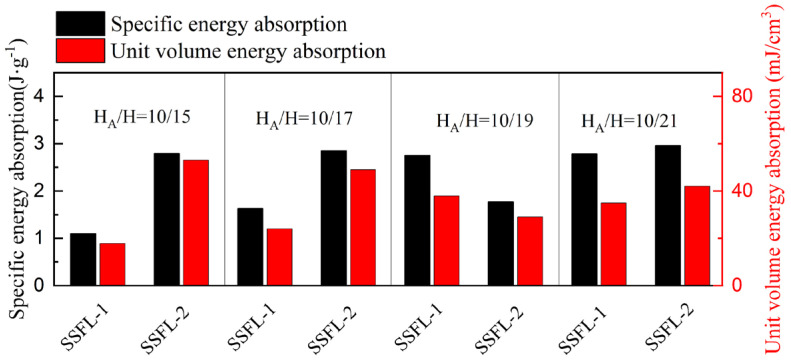
Comparison of specific energy absorption and unit volume energy absorption of SSFLs (a = 2, t = 1) in different H_A_/H.

From the stress–strain curves, the first bearing strengths of the SSFLs in different H_A_/H ratios are almost the same, indicating that the SSFLs reach the material limit. When H = 15, the greatest strengthening by the secondary fractal is obtained. The third bearing strength can reach two times the first bearing strength, reaching 0.2 MPa. Consequently, implementing secondary fractals and increasing the ratio of H_A_/H are more cost-effective strategies for slender strut structures. This is also true in terms of unit volume energy absorption, as shown in Figure 14.

**Figure 15:**
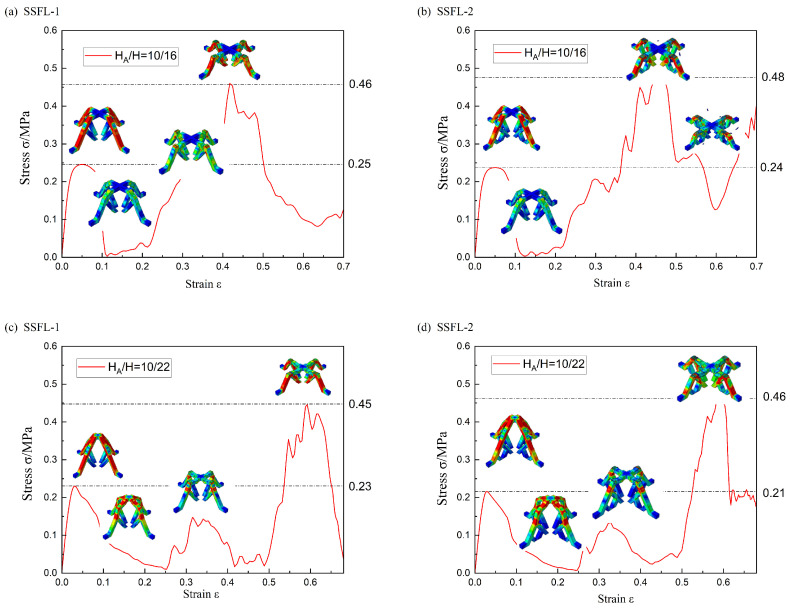
The deformation and stress–strain curves of the SSFLs (a = 2, t = 2) in different H_A_/H: (**a**) SSFL-1 in H_A_/H = 10/16; (**b**) SSFL-2 in H_A_/H = 10/16; (**c**) SSFL-1 in H_A_/H = 10/22; (**d**) SSFL-2 in H_A_/H = 10/22.

**Figure 16:**
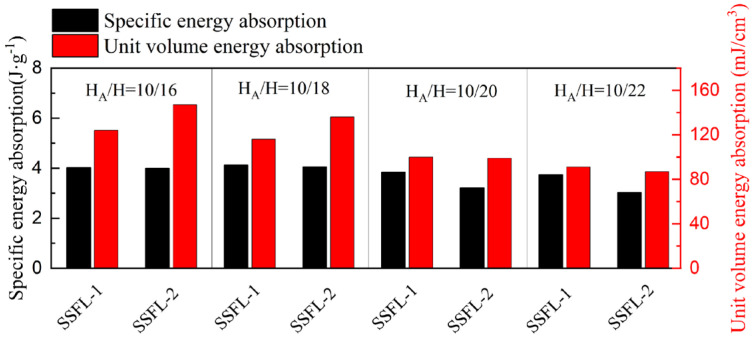
Comparison of specific energy absorption and unit volume energy absorption of SSFLs (a = 2, t = 2) in different H_A_/H.

When a = 2 and t = 2, the deformation modes and stress–strain curves of the SSFL-1 and SSFL-2 are similar for different values of H_A_/H, as shown in Figure 15. When H_A_/H = 10/16, the initial bending occurs in the trunk near the bifurcation point and there is a peak platform in the stress–strain curve. When H_A_/H = 10/22, the elongation of the trunk causes the failure to occur away from the trunk and causes the peak in the stress–strain to be sharper and lower than the SSFL in H_A_/H = 10/16. Overall, the secondary strengths of the SSFL-2s are slightly greater than those of the SSFL-1s. The unit volume energy absorption increases as the ratio of H_A_/H increases, and the SSFL-2s perform better, as shown in Figure 16.

Therefore, for the SSFL in (a = 2, t = 2), it is more beneficial to shorten the height and increase the fractal ratio.

## 5. Conclusions

In this paper, sandwich structures with tree-like fractal lattice cores at different orders were proposed. Quasi-static compression experiments and finite element simulations were performed to investigate the mechanical behaviors and energy absorption mechanisms of the sandwich structures. In addition, the influence of geometric parameters was also discussed to obtain the following conclusions:

1. Fractal design is a sufficient way to realize multiple bearings’ function and improve energy absorption by a dozen times. For the SSFL-1, the secondary bearing strength is 1.2 times the initial bearing strength, and the specific energy absorption is 3 times higher than the SSPL. For the SSFL-2, the secondary bearing strength is two times the initial bearing strength, and the specific energy absorption is four times higher than the SSPL.

2. The geometry of the cross-section is crucial to the mechanical capacities of the SSFLs. For slender structures with small cross-sectional dimensions, the fractal design not only realizes multiple bearings but also significantly improves the initial strength of the SSPL. For thicker struts, the fractal design helps the structure to gain higher secondary strength.

3. The different fractal ratios of H_A_/H will significantly change the deformation mode of the SSFLs, especially in slender structures. For slender struts, reducing the trunk length and adopting a second-order fractal design is an effective way to increase the bearing and energy absorption capacities. For thicker struts, increasing the fractal ratio (H_A_/H) is an effective way to increase the energy absorption capacity.

The design strategy proposed in this study provided a new opportunity to improve the energy absorption of composite sandwich structures with lattice cores by combining controllable deformation mechanisms and adjustable mechanical properties. With the assistance of advanced additive manufacturing techniques, it will play an important role in engineering applications. To assess the robustness of the structural performance, a significant number of experiments and statistical analyses are essential, which will be the focus of the next study.

## Figures and Tables

**Table 1:** Parameters of 3D-printed sandwich structures with fractal lattice cores.

Group	L/mm	L_1_/mm	L_2_/mm	a/mm	H/mm	t/mm	H_A_/mm	H_B_/mm	Relative Density/%
SSPL	40	4.5	4.5	2	20	2	-	-	1.46
SSFL-1	40	4.5	4.5	2	20	2	10	-	1.98
SSFL-2	40	4.5	4.5	2	20	2	10	4	2.35

**Table 2:** The true plastic stress–strain data of PA dog-bone.

Plastic Strain	0	0.001	0.002	0.003	0.004	0.005	0.006	0.018
Plastic stress/MPa	15	20.5	23.9	25.9	27.5	28.7	29.8	30.4

**Table 3:** Experimental, simulated, and theoretical compression strength comparisons of SSPL and SSFLs.

	Actual *σ*_i_	Simulated *σ*_i_a	Error 1	Calculation *σ*_i_	Error 2	Actual *σ* _ii_	Simulated *σ* _ii_	Error 1	Calculation *σ*_ii_	Error 2
SSPL	0.2	0.22	0.1	0.25	0.25	-	-	-	-	-
SSFL-1	0.2	0.21	0.05	0.25	0.25	0.25	0.29	0.16	0.5	0.71
SSFL-2	0.24	0.2	0.17	0.25	0.04	0.39	0.41	0.05	0.5	0.28

Strength *σ* is measured in MPa; σ_i_ and σ_ii_ are the initial and the secondary compressive strength of structures, respectively.

**Table 4:** Energy absorption of SSPL and SSFLs.

	Actual Total Energy Absorption/mJ	Unit Volume Energy Absorption (mJ/cm^3^)	Specific Energy Absorption (J·g^−1^)
SSPL	1138	11	0.95
SSFL-1	7105	74	2.84
SSFL-2	11063	117	3.79

**Table 5:** Simulation model designators of SSPL and SSFLs with different a and t.

	Group A (a = 1, t = 1 )	Group B (a = 1, t = 2)	Group C (a = 2, t = 1)	Group D (a = 2, t = 2)
SSPL	A_0_	B_0_	C_0_	D_0_
SSFL-1	A_1_	B_1_	C_1_	D_1_
SSFL-2	A_2_	B_2_	C_2_	D_2_

## Data Availability

The original contributions presented in this study are included in the article. Further inquiries can be directed to the corresponding author.
